# Effects of entinostat, quisinostat, and tubastatin-A on alcohol consumption in male high ethanol consuming rats

**DOI:** 10.3389/freae.2024.1503093

**Published:** 2025-02-03

**Authors:** Sheketha R. Hauser, Laura B. Ferguson, Tiebing Liang, Erin E. Jarvis, R. Dayne Mayfield, Richard L. Bell

**Affiliations:** ^1^ Department of Psychiatry, Indiana University School of Medicine, Indianapolis, IN, United States; ^2^ Paul and Carole Stark Neurosciences Research Institute, Indiana University School of Medicine, Indianapolis, IN, United States; ^3^ Waggoner Center for Alcohol and Addiction Research, University of Texas at Austin, Austin, TX, United States; ^4^ Department of Neuroscience, University of Texas at Austin, Austin, TX, United States; ^5^ Department of Gastroenterology, Indiana University School of Medicine, Indianapolis, IN, United States

**Keywords:** alcohol, alcohol-preferring P rats, high alcohol drinking line-1 HAD-1 rats, entinostat, quisinostat, tubastatin-A, epigenetics, HDAC inhibitors

## Abstract

**Background:**

The interaction between genetics, epigenetics, and the environment plays a key role in the development of alcohol use disorder (AUD). Pharmacological treatments targeting histone deacetylases (HDACs) suggest that HDAC inhibitors (HDACi) may be potential pharmacotherapeutic treatments for AUD. The objective of the current study was to test the effects of different HDACi on ethanol intake in two rat lines selectively bred for high ethanol-consumption.

**Method:**

Adult naïve male high alcohol drinking line 1 (HAD1) or alcohol-preferring (P) rats were given continuous 24-h, 3-bottle, free-choice access to 15%, 30% ethanol concurrently with water for 8 weeks prior to testing entinostat (selective HDAC1i and HADC3i, 0, 1.25, 2.5, 5 mg/kg, i. p.), quisinostat (pan HADCi, 0, 0.5, 1.0, 2.0 mg/kg, i. p.), or tubastatin-A (selective HDAC6i, 0, 1.25, 2.5, 5 mg/kg, i. p.) over 4-5 consecutive days.

**Results:**

In HAD1 rats, entinostat reduced 2-, 4-, and 24-h ethanol intake across the 2^nd^ – 5^th^ test days; while, in P rats, entinostat’s effect was primarily seen at the 24-h time-point, at the highest dose and only across three test days. The high dose of quisinostat effectively reduced 24-h ethanol intake across the 1^st^—4^th^ test days in HAD1 rats but was ineffective in P rats. Tubastatin-A did not alter ethanol intake in either rat line.

**Conclusion:**

Overall, the results confirmed that a pan and a more selective (HDAC1 and HDAC3) HDACi effectively reduced ethanol intake in HAD1, while only the more selective HDACi reduced ethanol intake in P rats. Inhibition of HDAC6 does not appear to regulate ethanol intake in HAD1 or P rats.

## Introduction

Chronic alcohol use is a leading cause of preventable death in the United States (Center for Disease Control, 2025). It causes or exacerbates health and social problems, increases mortality rate, and costs society nearly a quarter trillion dollars each year (c.f., National Institute of Alcohol Abuse and Alcoholism [NIAAA], 2023; Rehm and Imtiaz, 2016). There are only a limited number of FDA-approved pharmacotherapies for alcohol use disorder (AUD) (e.g., Koob and Mason, 2016). Therefore, it is critical that we identify novel, efficacious pharmacotherapeutics for AUD. Genetic studies indicate that individuals with a family history positive (FHP) for alcoholism are more susceptible to developing AUD than family history negative (FHN) subjects (e.g., Lai et al., 2022). However, not every FHP individual develops an AUD, and FHN individuals can often develop AUD. Environmental risk factors such as other drugs of abuse, stress, history of child abuse, poverty, etc., can also contribute to the development of AUD (Deeken et al., 2020). Thus, the interaction of genes and environmental risk factors combine to enhance the risk for developing an AUD (Nieratschker, Batra, and Fallgatter, 2013). In addition, environmental risk factors can alter epigenetics, resulting in changes in phenotypes by modifying the chromatin structure or controlling gene expression without affecting the underlying DNA sequence (e.g., Peedicayil, 2023). Therefore, interactions between genetics, epigenetics, and environmental risk factors, all play a role in the development of AUD (e.g., Nieratschker, Batra, and Fallgatter, 2013). A commonly studied epigenetic modification is induced by histone (de)acetylation processes, which generally increase genetic transcription or accelerate rates of protein production (e.g., Moore, 2017; Pandey and Bohnsack, 2020). Clinical and pre-clinical studies have shown that alcohol exposure can alter histone acetylation and deacetylation via histone acetyltransferases (HATS), histone deacetylase (HDAC) enzymes, and other epigenetic processes (López-Moreno et al., 2015; Pandey et al., 2017; De Carvalho et al., 2021).

Epigenetic modifications are stable but can be reversed. For example, administrating HDAC inhibitors (HDACi) can reverse some of these epigenetic modifications, such as (de)acetylation. HDACs are repressors of gene transcription by deacetylating histone proteins, leading to a transcriptionally repressed chromatin structure. HDACs are divided into zinc-dependent and nicotinamide-adenine-dinucleotide (NAD)-dependent sirtuins (Hull et al., 2016). The HDAC class I family (HDACs 1, 2, 3, and 8), class IIa and IIb families (HDACs 4, 5, 6, 7, 9, and 10), as well as class IV family (HDAC 11) are zinc-dependent, and widely expressed in the brain (Hull et al., 2016; Palmisano and Pandey, 2017). In particular, isoforms of class I, II, and IV HDACs are expressed in brain neurons (Broide et al., 2007). There are low levels of HDACs found in astrocytes and HDACS 2, 3, 4, 5, and 11 are expressed in oligodendrocytes (Graff and Tsai, 2013). Class I HDACs, except for HDCAC six (only cytoplasm), are located in both the nucleus and cytoplasm (Gibson and Murphy, 2010). As noted above, HDACs are implicated in the development of alcohol dependence, ethanol-seeking, and other ethanol/drug-associated behaviors (Werner et al., 2021; Palmisano and Pandey, 2017; Pandey et al., 2017; Kamat et al., 2016). *In vivo* ethanol exposure and ethanol withdrawal can increase the expression of Class I HDACs, particularly HDAC2, in rodents (Arora et al., 2013; Pandey et al., 2015; Bohnsack et al., 2018; Chen et al., 2019), while *in vitro* exposure to alcohol can upregulate gene and protein expression levels of HDAC2 in human cells (Agudelo et al., 2011; Agudelo et al., 2016). Agudelo and colleagues (2016) found that all class I HDACs (1, 2, 3, and 8) when examined *ex vivo* in human primary monocyte-derived dendritic cells, from alcohol users, displayed significantly higher HDAC-associated RNA and protein levels compared to their controls (Agudelo et al., 2016).

The first generation pan HDACi (i.e., an inhibitor of Class I and Class II HDACs), trichostatin A (TSA), is a common HDACi used to examine the involvement of HDACs in excessive ethanol intake by rodents. TSA reduces voluntary ethanol drinking in alcohol-preferring (P) rats, to a greater extent than that observed in alcohol-nonpreferring (NP) rats (Sakharkar et al., 2014). It also reduced ethanol-drinking by outbred Wistar rats using an intermittent 2-bottle choice protocol (Bohnsack et al., 2018). These findings suggest that TSA attenuates ethanol consumption in both genetically selected and non-selected rodents. Clinically, HDAC inhibitors are used as anticancer treatments. However, pan-HDAC inhibitors lead to more adverse side effects such as diarrhea, nausea, vomiting, fatigue, thrombopenia, and neutropenia (c.f., Hontecillas-Prieto et al., 2020). These adverse side effects are reduced when treated with more selective HDACi’s (c.f., Hontecillas-Prieto et al., 2020). To date, there have been no published research examining the effects of entinostat (predominantly an HDAC1 and HADC3 inhibitor), quisinostat (second generation pan HADCi), and tubastatin-A (selective HDAC6 inhibitor) on ethanol consumption in rodents.

The risk for developing an AUD can be modeled through rodent bidirectional selective breeding for high ethanol consumption and preference vs. low ethanol consumption and nonpreference. The P and high alcohol drinking (HAD1) rat lines rats satisfy criteria put forth for an animal model of alcoholism (c.f., Bell et al., 2012; 2017; McBride and Li, 1998; McBride et al., 2014; Murphy et al., 2002). Research on genetic factors contributing to a vulnerability for developing high ethanol-consuming behavior have paralleled findings with FHP, *versus* control, individuals (e.g., Li et al., 1993; alpha-synuclein; Liang et al., 2003). Both P and HAD rats achieve pharmacologically, often exceeding that associated with binge-drinking (80 mg%), relevant blood alcohol concentrations under limited access, continuous access, and relapse-like conditions (Bell et al., 2006; 2011; 2012; 2014; Dhaher et al., 2012; Murphy et al., 1986; 2002). Therefore, the objectives of the present study were to examine the effects of entinostat, quisinostat, and tubastatin-A on ethanol drinking by adult male HAD1 and P rats.

## Materials and methods

### Animals

Only male rats were used in the current study. The subjects were ethanol-naïve P and HAD1 rats. Subjects were at least 60 days old at the start of the experiments. All rats received free access to standard laboratory chow (Teklad 2,918X; Envigo, Indianapolis, IN, United States) and water throughout the experiments. The animals were maintained on a 12/12-h reverse dark/light cycle (lights off at 1,100 h). Subjects were housed in a temperature- (21°C) and humidity- (50%) controlled vivarium. All research protocols were approved by the Institutional Animal Care and Use Committee (IACUC) of the Indiana University School of Medicine (Indianapolis, IN, United States) and are in accordance with the guidelines of the IACUC of the National Institute on Alcohol Abuse and Alcoholism (NIAAA), National Institute on Drug Abuse (NIDA), National Institutes of Health (NIH), and the Guide for the Care and Use of Laboratory Animals (Research Institute for Laboratory Animal Research, 2011).

### Test compounds

The ethanol solution was prepared as 15% and 30% v/v in tap water from 190-proof ethanol (Fischer Scientific, Waltham, MA, United States). Entinostat (selective HDAC1-HADC3 inhibitor; SelleckChem, Houston, TX, United States), quisinostat (second generation pan-HDAC inhibitor; SelleckChem, Houston, TX, United States), and tubastatin-A (selective HDAC6 inhibitor; SelleckChem, Houston, TX, United States) were mixed daily with 0.25% Tween (Sigma-Aldrich, St. Louis, MO, United States) in sterile saline for a volume of 1.5 mL/kg body weight. Quisinostat (N = 60; n = 10/dose for HAD1 rats; n = 5/dose for P rats) was administered intraperitoneally (i.p.) at the following doses: 0, 0.5, 1.0, or 2.0 mg/kg. These doses were selected to approximate those previously reported for TSA, another pan-HDACi, that reduced ethanol drinking in P rats (Sakharkar et al., 2014). Entinostat (N = 72; n = 10/dose for HAD1 rats; n = 8/dose for P rats) and tubastatin-A (N = 80; n = 10/dose for HAD1 rat; n = 10/dose for P rats) were administered i. p. at the following doses: 0, 1.25, 2.5, or 5.0 mg/kg. The entinostat doses were selected to approximate those previously reported by Warnault et al. (2013), and similar doses were used for tubastatin-A. The vehicle consisted of 0.25% Tween in sterile saline.

### Continuous 24-h (3-bottle choice) access to ethanol

The rats were individually housed in hanging stainless steel wire-mesh cages (with individual Plexiglas platform in the cages) for 3-bottle choice (3BC) ethanol (15% and 30%, concurrently) drinking access. The rats were allowed to consume 15% and 30% ethanol with water for 8 weeks of 24-h continuous access drinking (Figure 1, experimental timeline created with BioRender^®^).

**FIGURE 1:**
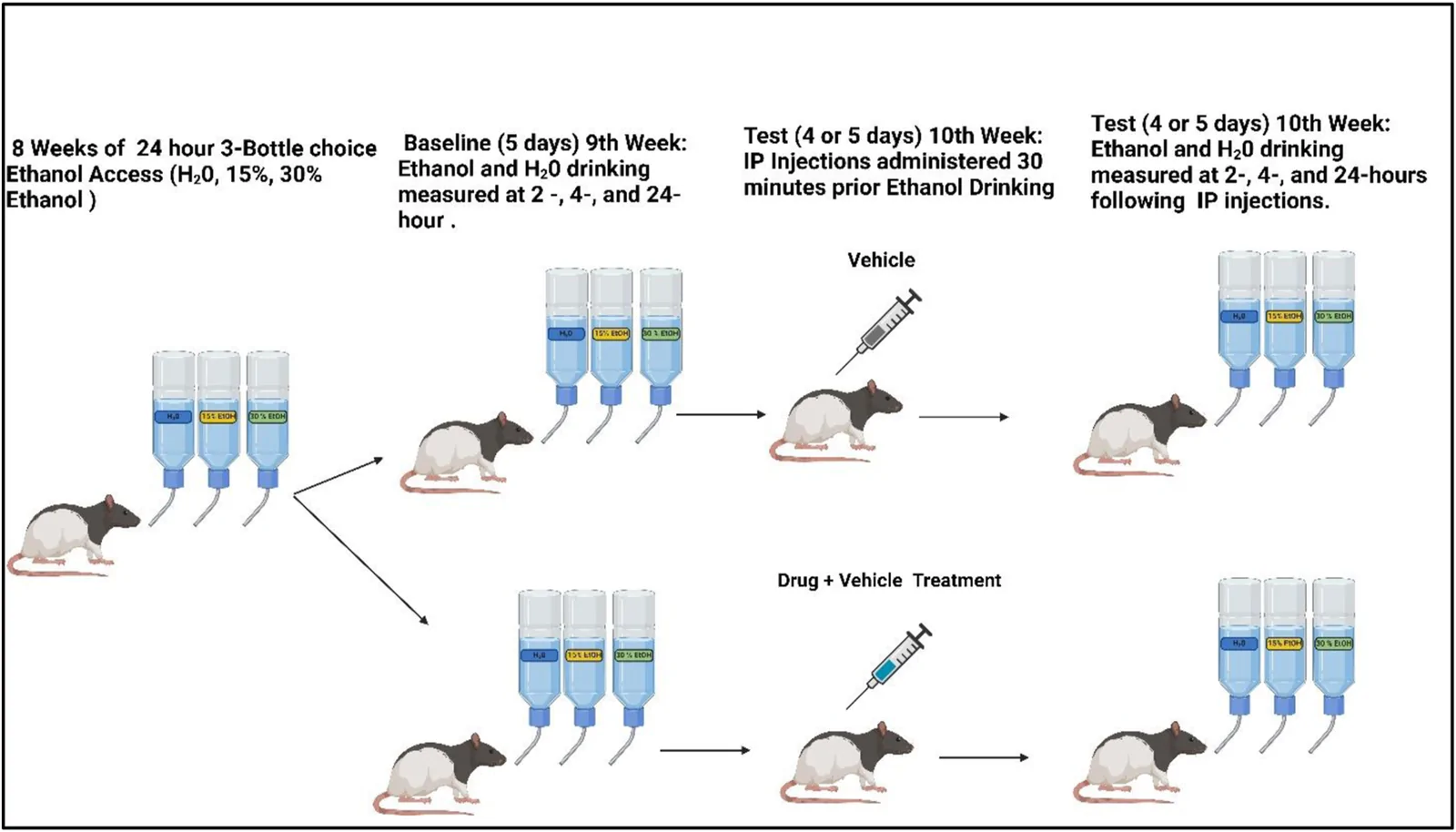
Depicts the experimental timeline.

### Compound testing procedures

The effects of entinostat, quisinostat, or tubastatin-A on ethanol intake in HAD1 and P rats were examined at 2-h (1300 h), 4-h (1500 h), and 24-h (∼1045 h) post-injection (injection at 1030–1100 h). A different cohort of animals was used for each test compound to avoid any carry-over effects. The rats were randomly assigned to treatment groups and dose group membership was balanced using the last 5 days of ethanol drinking, before the week of testing, as the baseline. During the test week (Monday-Friday), ethanol, water, food, and body weight measures were collected at 1,000 h (1 h prior to lights out). Entinostat, quisinostat, or tubastatin-A were administered daily at ∼1,030 h (30 min prior to lights out) for four or five consecutive days. P rats were treated with quisinostat for only four test days due to the limited availability of the test compound (to ensure drugs from the same lot number were used). Tubastatin-A was tested for 4 days in P and HAD1 rats due to the limited availability of the test compound (to ensure drugs from the same lot number were used). Ethanol and water access were returned at 1,100 h (lights out) on test days.

## Data analyses and statistics

Ethanol and water intakes were determined as the difference in the weight of glass bottle, and its contents, prior to and after each time-point (2-, 4-, and 24-h). Ethanol intake was then converted to g of absolute (i.e., corrected for ethanol’s specific gravity) ethanol consumed/kg of body weight/unit of time measurement (g/kg/time-period), water-intake was measured as ml/time-period, food-intake was measured as g/kg body weight, and body weight measured in grams. IBM SPSS version 29 was used to analyze data, and GraphPad version 10.3.1 was used to graph the data (mean ± SEM). Data were analyzed with mixed ANOVAs, with repeated measures for time-point and test day, and appropriate simple main effects with one-way ANOVAs, followed by Dunnett’s *t*-test for planned comparisons (i.e., dose vs. control). P-values <0.05 were considered statistically significant.

## Results

### Effects of entinostat on ethanol intake

#### P rats

Two-hour ethanol intake. For the P rats, analysis of the 2-h ethanol intake data revealed significant main effects of Dose [F(3,28) = 2.938, p = 0.05; partial eta-squared = 0.239 with a power of 0.633] and Test Day [F(4,112) = 6.452, p < 0.001; partial eta-squared = 0.187 with a power of 0.989] (Figure 2). Planned comparison Dunnett t-tests revealed the middle dose of entinostat significantly reduced ethanol intake on the third test day.

**FIGURE 2:**
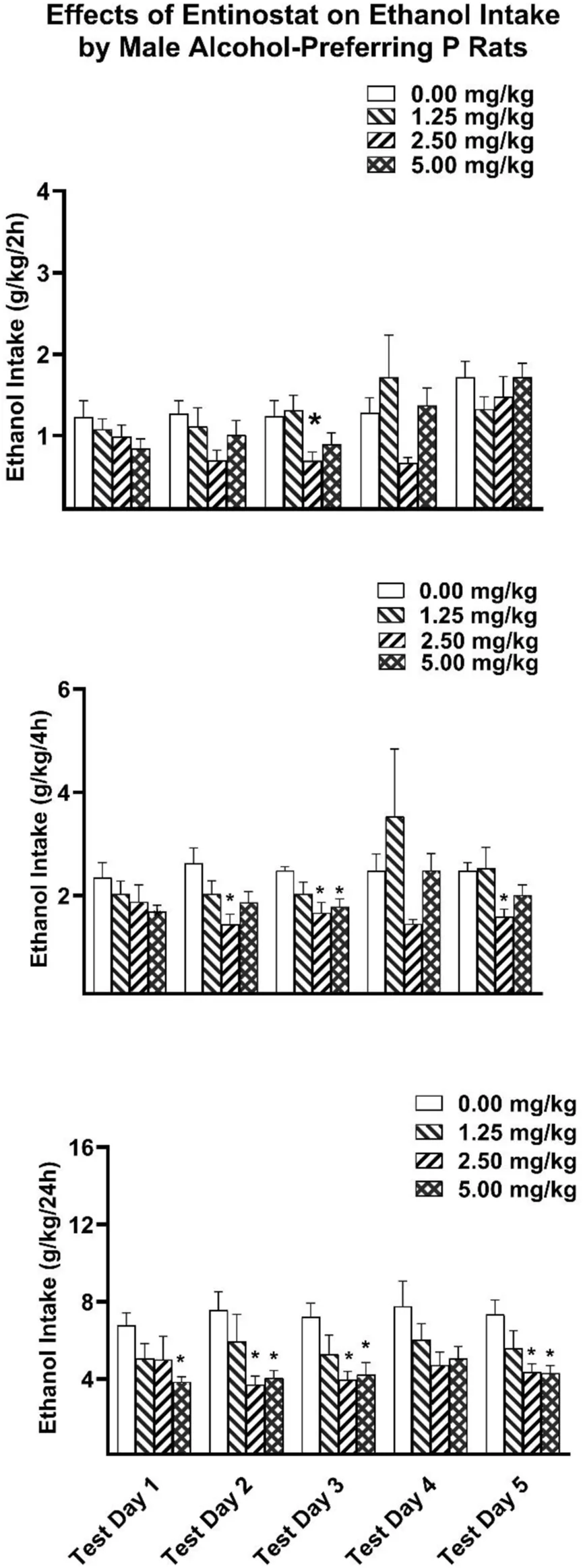
Depicts the mean (±SEM) ethanol intake (g/kg) in male P rats at (Upper Panel) 2-h, (Middle Panel) 4-h, and (Lower Panel) 24-h time points following i. p. administration 0, 1.25, 2.5, 5 mg/kg of entinostat. *(*p* < 0.05) Indicates that entinostat significantly reduced ethanol compared to control values in male P rats.

Four-hour ethanol intake. Analysis of the 4-h ethanol intake revealed a significant main effect of Dose [F(3,28) = 3.814, p = 0.021; partial eta-squared = 0.290 with a power of 0.759]. Planned comparison Dunnett t-tests revealed the middle dose of entinostat significantly reduced ethanol intake on the second, third, and fifth test days, with the highest dose of entinostat significantly reducing ethanol intake on the third test day.

Twenty 4-h ethanol intake. Analysis of the 24-h ethanol intake revealed a significant main effect of Dose [F(3,28) = 4.648, p = 0.009; partial eta-squared = 0.332 with a power of 0.844]. Planned comparison Dunnett t-tests revealed that the two higher doses of entinostat significantly reduced ethanol intake on the second, third, and fifth test days, with the highest dose of entinostat also reducing ethanol intake on the first test day.

#### HAD1 rats 

Two-hour ethanol intake. For the HAD1 rats, analysis of the 2-h ethanol intake data revealed significant main effects of Dose [F(3,36) = 23.857, p < 0.001; partial eta-squared = 0.665 with a power of 0.999] and test day [F(4,144) = 20.983, p < 0.001; partial eta-squared = 0.368 with a power of 0.999] (Figure 3). Planned comparison Dunnett t-tests revealed on the first test day the lowest and highest doses of entinostat significantly reduced ethanol intake. All three doses significantly reduced ethanol intake on the second through fifth test days.

**FIGURE 3:**
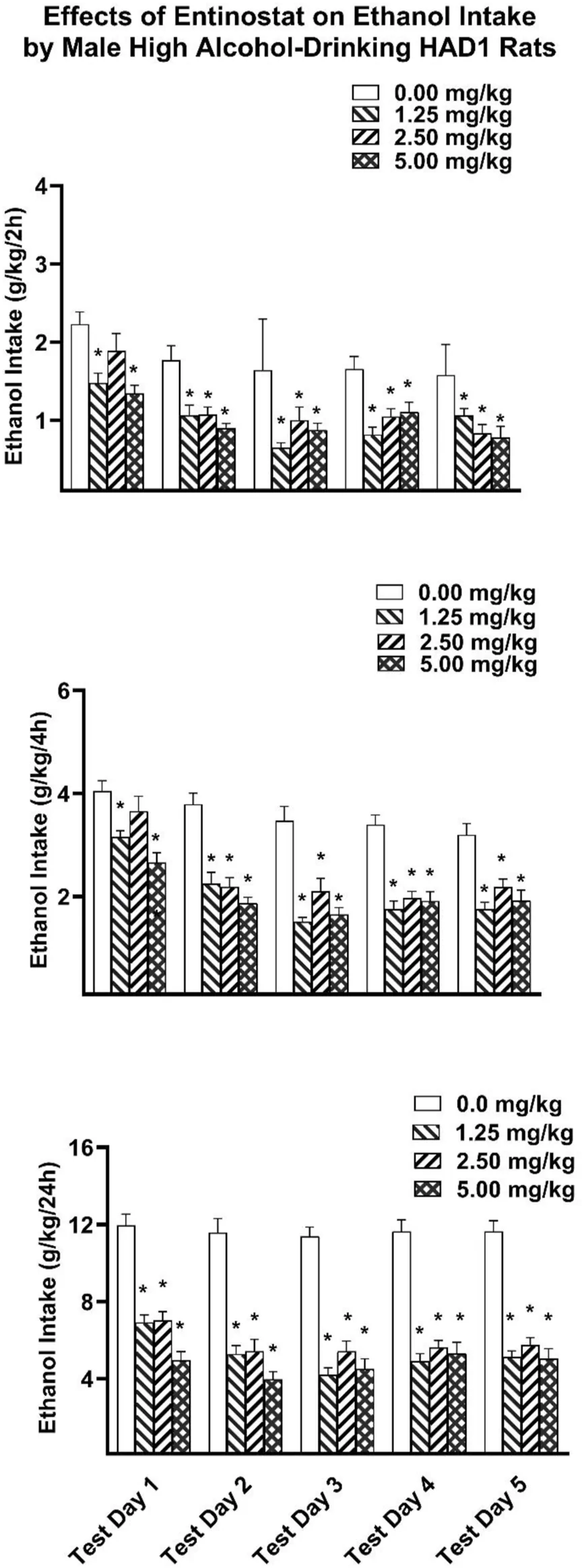
Depicts the mean (±SEM) ethanol intake (g/kg) in male HAD1 rats at (Upper Panel) 2-h, (Middle Panel) 4-h, and (Lower Panel) 24-h time points following i. p. administration 0, 1.25, 2.5, 5 mg/kg of entinostat. *(*p* < 0.05) Indicates that entinostat significantly reduced ethanol compared to control values in male HAD1 rats.

Four-hour ethanol intake. Analysis of the 4-h ethanol intake revealed a significant Dose × Test Day interaction [F(12,144) = 2.530, p = 0.005; partial eta-squared = 0.174 with a power of 0.968] as well as main effects of Dose [F(3,36) = 33.928, p < 0.001; partial eta-squared = 0.739 with a power of 0.999] and Test Day [F(4,144) = 39.123, p < 0.001; partial eta-squared 0.521 with a power of 0.999]. Planned comparison Dunnett t-tests revealed the lowest and highest doses of entinostat significantly reduced ethanol intake on the first test day. All three doses significantly reduced ethanol intake on the second through fifth test days.

Twenty 4-h ethanol intake. Analysis of the 24-h ethanol intake revealed a significant Dose × Test Day interaction [F(12,144) = 3.572, p < 0.001; partial eta-squared = 0.229 with a power of 0.997] as well as main effects for Dose [F(3,36) = 53.422, p < 0.001; partial eta-squared = 0.817 with a power of 0.999] and Test Day [F(4,144) = 15.560, p < 0.001; partial eta-squared = 0.302 with a power of 0.999]. Planned comparison Dunnett t-tests revealed that all three doses of entinostat significantly reduced ethanol intake across all five Test Days.

### Effects of entinostat on water intake

#### P rats

For the P rats, analysis of the 2-h water intake data revealed no significant Dose (p > 0.05) effects (Supplementary Figure S1). Analysis of the 4-h water intake revealed no significant Dose (p > 0.05) effects. Analysis of the twenty-four -hour water intake revealed a significant main effect for Dose [F(3,28) = 5.629, p = 0.004; partial eta-squared = 0.376 with a power of 0.910].

For the 2-h water intake, the planned comparison Dunnett t-tests revealed the middle dose of Entinostat significantly increased water intake on the third test day, whereas there were no significant Dose effects for the 4-h water intake. For the 24-h water intake, planned comparison Dunnett t-tests revealed that the lowest entinostat Dose increased water intake on the second test day, the lowest and mid doses of entinostat significantly increased water intake on the third test day, and all entinostat doses significantly increased water intake on the fourth test day.

#### HAD1 rats

For the HAD1 rats, analysis of the 2-h water intake data revealed a main effect of Dose [F(3,36) = 3.790, p = 0.018; partial eta-squared = 0.240 with a power of 0.770] (Supplementary Figure S2). Analysis of the 4-h water intake revealed a significant Dose × Test Day interaction [F(12,144) = 2.789, p = 0.002; partial eta-squared = 0.189 with a power of 0.982] as well as a significant Dose main effect [F(3,36), p = 0.028; partial eta-squared = 0.221 with a power of 0.719]. Analysis of the 24-h water intake revealed a significant Dose × Test Day interaction [F(9,108) = 2.124, p = 0.033; partial eta-squared = 0.150 with a power of 0.856] as well as a main effect for Dose [F(3,36) = 11.306, p < 0.001; partial eta-squared = 0.485 with a power of 0.998].

Two-hour water intake: planned comparison Dunnett t-tests revealed the highest dose of entinostat significantly increased water intake on the second and the third test days. In addition, the lowest entinostat dose significantly increased water intake on the fourth test day. Four-hour water intake: the planned comparison Dunnett’s t-tests revealed the highest entinostat Dose significantly increased water intake on the first and second test days and the lowest entinostat Dose significantly increased water intake on the third and fourth test days. Twenty-four-hour water intake: planned comparison Dunnett’s t-tests revealed that the lowest and highest entinostat Dose significantly increased water intake on the first test day. In addition, all three entinostat doses significantly increased water intake on the second, third, and fourth test days.

### Effects of entinostat on body weight and food intake

#### P rats

For the P rats, analysis of the body weight revealed a significant Dose × Test Day interaction [F(12,112) = 7.355, p < 0.001; partial eta-squared = 0.441 with a power of 0.999] but the Dose main effect was not significant (Supplementary Figure S7). Analysis of the food intake data revealed a significant Dose main effect [F(3,28) = 7.445, p < 0.001; partial eta-squared = 0.444 with a power of 0.971], which was primarily driven by the high entinostat dose significantly decreasing food intake across test days 2 through 4.

#### HAD1 rats

For the HAD1 rats, analysis of the body weight revealed a significant Dose × Test Day interaction [F(12,144) = 13.851, p < 0.001; partial eta-squared = 0.536 with a power of 0.999] but the Dose main effect was not significant (Supplementary Figure S8). Analysis of the food intake data revealed a significant Dose main effect [F(3,36) = 6.853, p < 0.001; partial eta-squared = 0.363 with a power of 0.963], which was primarily driven by the high entinostat Dose significantly decreasing food intake across Test Days 2 through 4.

### Effects of quisinostat on ethanol intake

#### P rats

For the P rats, analysis of the 2-h ethanol intake data revealed significant main effect for Test Day [F(3,48) = 5.678, p = 0.002; partial eta-squared = 0.262 with a power of 0.929] (Figure 4). Analysis of the 2-h ethanol intake data did not reveal any significant Dose effects. Analysis of the 4-h and twenty-four ethanol intakes revealed that there were no significant interactions or main effects of dose and test day.

**FIGURE 4:**
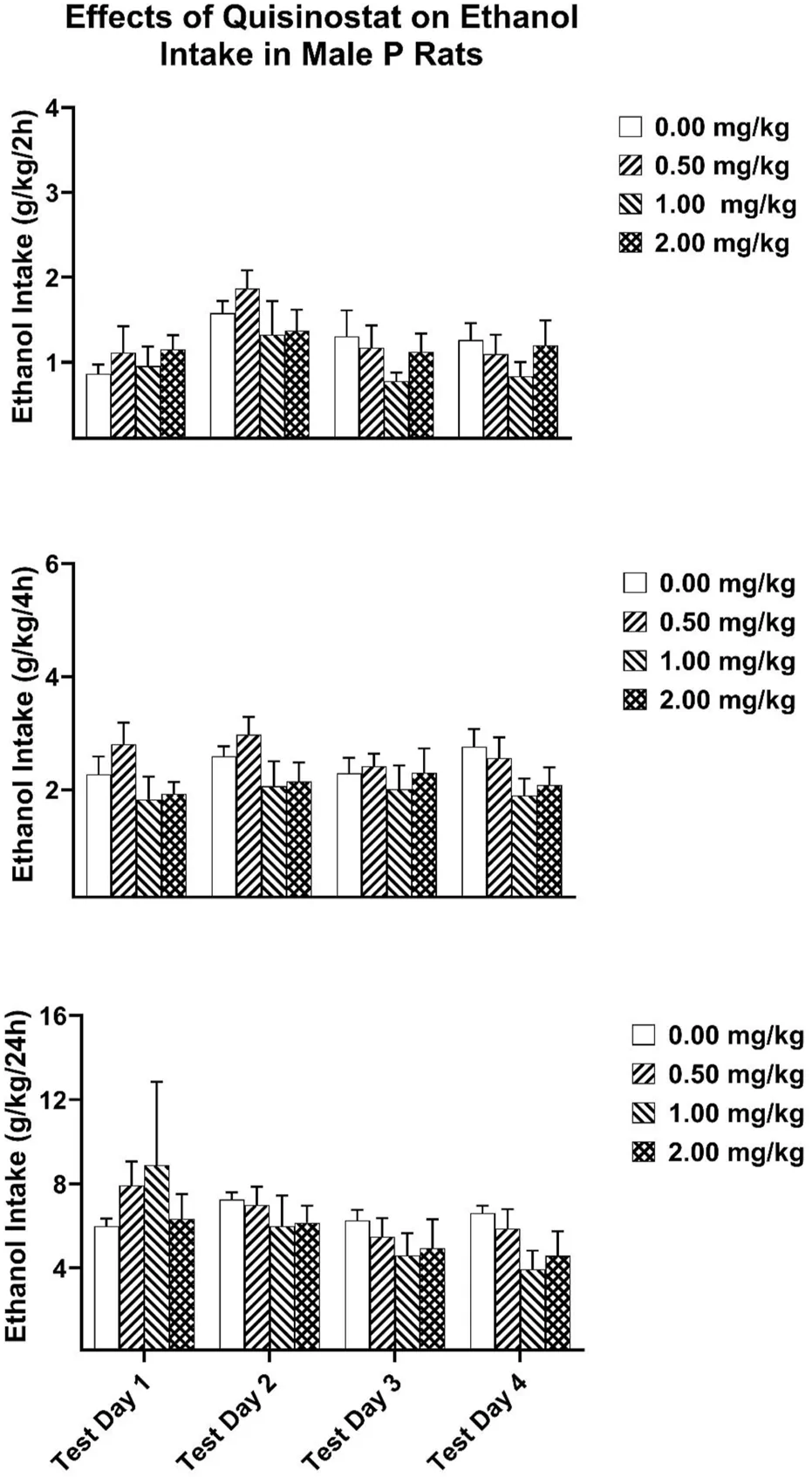
Depicts the mean (±SEM) ethanol intake (g/kg) in male P rats at (Upper Panel) 2-h, (Middle Panel) 4-h, and (Lower Panel) 24-h time points following i. p. administration 0, 0.5, 1, 2 mg/kg of quisinostat.

#### HAD rats

For the HAD1 rats, analysis of the 2-h ethanol intake data revealed a significant Dose × Test Day interaction [F(9,108) = 4.601, p < 0.001; partial eta-squared = 0.277 with a power of 0.998] as well as significant main effects for Dose [F(3,36) = 9.702, p = 0.011; partial eta-squared = 0.447with a power of 0.995] and Test Day [F(3,108) = 20.921, p < 0.001; partial eta-squared = 0.368 with a power of 0.999] (Figure 5). Analysis of the 4-h ethanol intake revealed a significant Dose × Test Day interaction [F(9,108) = 5.456, p < 0.001; partial eta-squared = 0.313 with a power of 0.999] as well as main effects of Dose [F(3,36) = 5.157, p = 0.005; partial eta-squared = 0.301 with a power of 0.893] and Test Day [F(3,108) = 16.699, p < 0.001; partial eta-squared 0.317 with a power of 0.999]. Analysis of the 24-h ethanol intake revealed a significant Dose × Test Day interaction [F(9,108) = 8.779, p < 0.001; partial eta-squared = 0.422 with a power of 0.999] as well as main effects for Dose [F(3,36) = 13.980, p < 0.001; partial eta-squared = 0.5838 with a power of 0.999] and Test Day [F(3,108) = 37.792, p < 0.001; partial eta-squared = 0.477 with a power of 0.999].

**FIGURE 5:**
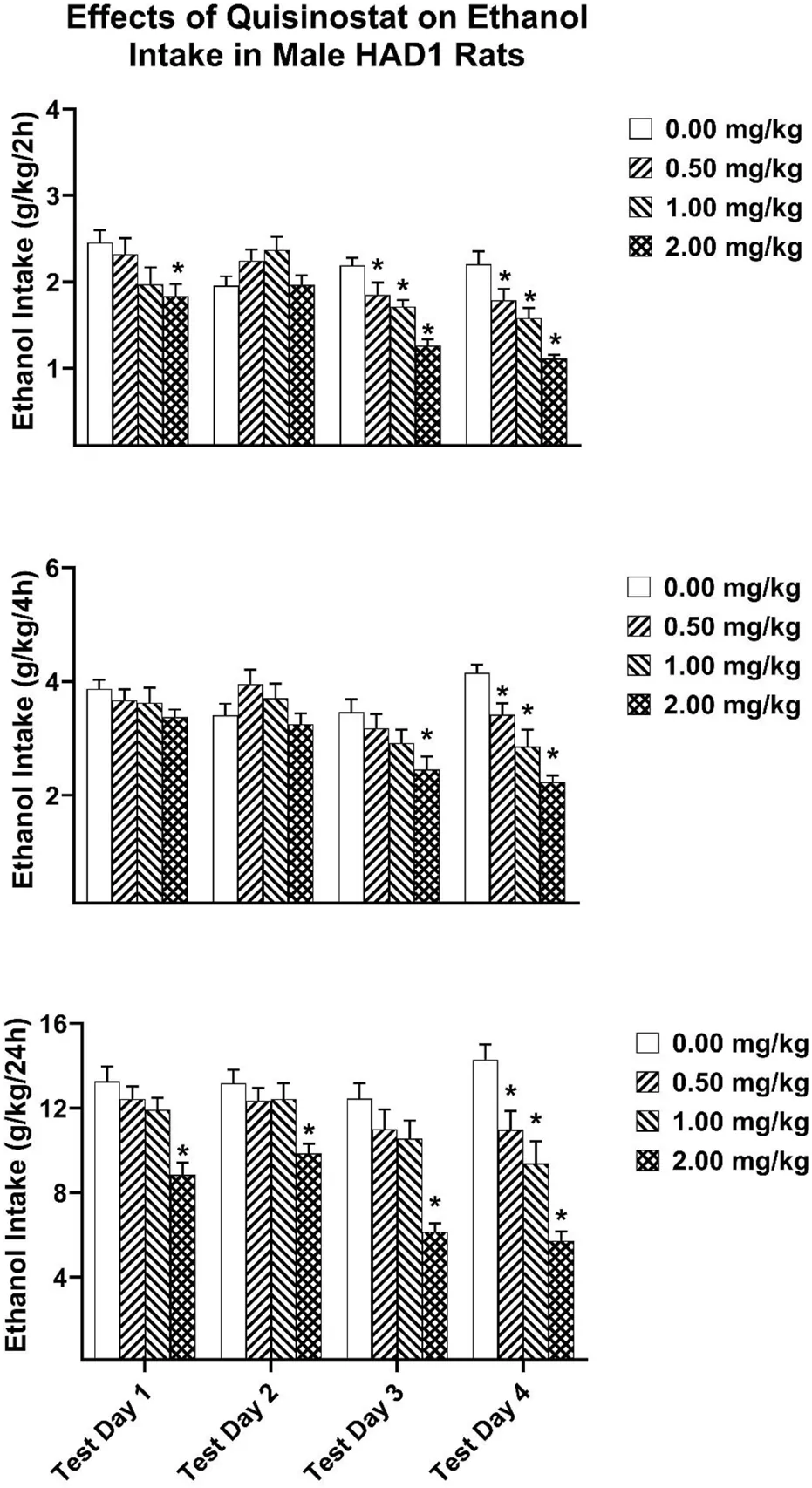
Depicts the mean (±SEM) ethanol intake (g/kg) in male HAD1 rats at (Upper Panel) 2-h, (Middle Panel) 4-h, and (Lower Panel) 24-h time points following i. p. administration 0, 0.5, 1, 2 mg/kg of quisinostat. *(*p* < 0.05) Indicates that quisinostat significantly reduced ethanol compared to control in male HAD1 rats.

Two-hour ethanol intake: planned comparison Dunnett t-tests revealed the highest dose of quisinostat significantly reduced ethanol on the first test day. All three doses significantly reduced ethanol intake on the third and fourth test days. Four-hour ethanol intake: planned comparison Dunnett t-tests revealed the highest dose of quisinostat significantly reduced ethanol intake on the third test day. All three doses significantly reduced ethanol intake on the fourth test day. Twenty-four-hour ethanol intake: planned comparison Dunnett t-tests revealed that the highest dose of quisinostat significantly reduced ethanol intake across all four test days. The lowest and mid-dose of quisinostat significantly reduced ethanol intake on the fourth test day.

### Effects of quisinostat on water intake

#### P rats

For the P rats, analysis of the 2-h water intake data revealed no significant Dose (p > 0.05) effects. Analysis of the 4-h water intake revealed no significant Dose (p > 0.05) effects (Supplementary Figure S3). Analysis of the 24-h water intake revealed a significant Dose × Test Day interaction [F(9,48) = 2.211, p = 0.037; partial eta-squared = 0.293 with a power of 0.834] as well as a main effect of Dose [F(3,16) = 5.442, p < 0.009; partial eta-squared = 0.505 with a power of 0.863]. For the Twenty-four hour water intake, the planned comparison Dunnett t-tests revealed that the highest quisinostat dose increased water intake on the first and fourth test days.

#### HAD1 rats

For the HAD1 rats, analysis of the 2-h water intake data revealed a significant Dose × Test Day interaction [F(9,108) = 2.778, p = 0.006; partial eta-squared = 0.188 with a power of 0.946] as well as a main effect of Dose [F(3,36) = 11.120, p < 0.011; partial eta-squared = 0.481 with a power of 0.998] (Supplementary Figure S4). Analysis of the 4-h water intake revealed a significant Dose × Test Day interaction [F(9,108) = 3.547, p < 0.001; partial eta-squared = 0.228 with a power of 0.985] as well as a significant Dose main effect [F(3,36) = 9.236, p < 0.001; partial eta-squared = 0.435 with a power of 0.993]. Analysis of the 24-h water intake revealed a significant Dose × Test Day interaction [F(9,108) = 3.655, p < 0.001; partial eta-squared = 0.233 with a power of 0.988] as well as a main effect for Dose [F(3,36) = 7.043, p < 0.001; partial eta-squared = 0.370 with a power of 0.967].

Two-hour water intake: planned comparison Dunnett t-tests revealed the highest dose of quisinostat significantly increased water intake on the second and fourth test days. Four-hour water intake: the planned comparison Dunnett’s t-tests revealed the highest quisinostat dose significantly increased water intake on the first through fourth test days. Twenty-four-hour water intake: planned comparison Dunnett’s t-tests revealed that the highest quisinostat dose significantly increased water intake on the first, third, and fourth test days.

### Effects of quisinostat on body weight and food intake

#### P rats

For the P rats, analysis of the body weight revealed a significant Dose × Test Day interaction [F(9,48) = 3.096, p = 0.005; partial eta-squared = 0.367 with a power of 0.949] but the Dose main effect was not significant (Supplementary Figure S9). Analysis of the food intake data revealed a significant Dose × Test Day interaction [F(9,48) = 2.989, p = 0.007; partial eta-squared = 0.359 with a power of 0.941]. There was also a Dose main effect [F(3,16) = 9.748, p < 0.001; partial eta-squared = 0.646 with a power of 0.987] and Test Day main effect [F(3,48) = 12.649, p < 0.001; partial eta-squared = 0.442 with a power of 0.999], which was primarily driven by the high quisinostat Dose significantly decreasing food intake across test days 2 through 4.

#### HAD1 rats

For the HAD1 rats, analysis of the body weight revealed a significant Dose × Test Day interaction [F(9,108) = 5.501, p < 0.001; partial eta-squared = 0.314 with a power of 0.999] but the Dose main effect was not significant (Supplementary Figure S10). Analysis of the food intake data revealed a significant Dose × Test Day interaction [F(9,108) = 5.475, p < 0.001; partial eta-squared = 0.313 with a power of 0.999] but the Dose main effect was not significant.

### Effects of tubastatin-A on ethanol intake

#### P rats

For the P rats, the Dose × Test Day x Test Time mixed ANOVA revealed a significant Dose × Test Time [F(6,70) = 2.848, p = 0.015; partial eta-squared = 0.196 with a power of 0.860] as well as a Test Day x Test Time [F(6,210) = 2.310, p = 0.035; partial eta-squared = 0.062 with a power of 0.794] interaction (Figure 6). Analysis of the two- and 4-h ethanol intake data did not reveal any significant Dose effects. Analysis of the 24-h ethanol intake only revealed a main effect of Dose that approached significance [F(3,35) = 2.762, p = 0.057; partial eta-squared = 0.191 with a power of 0.617].

**FIGURE 6:**
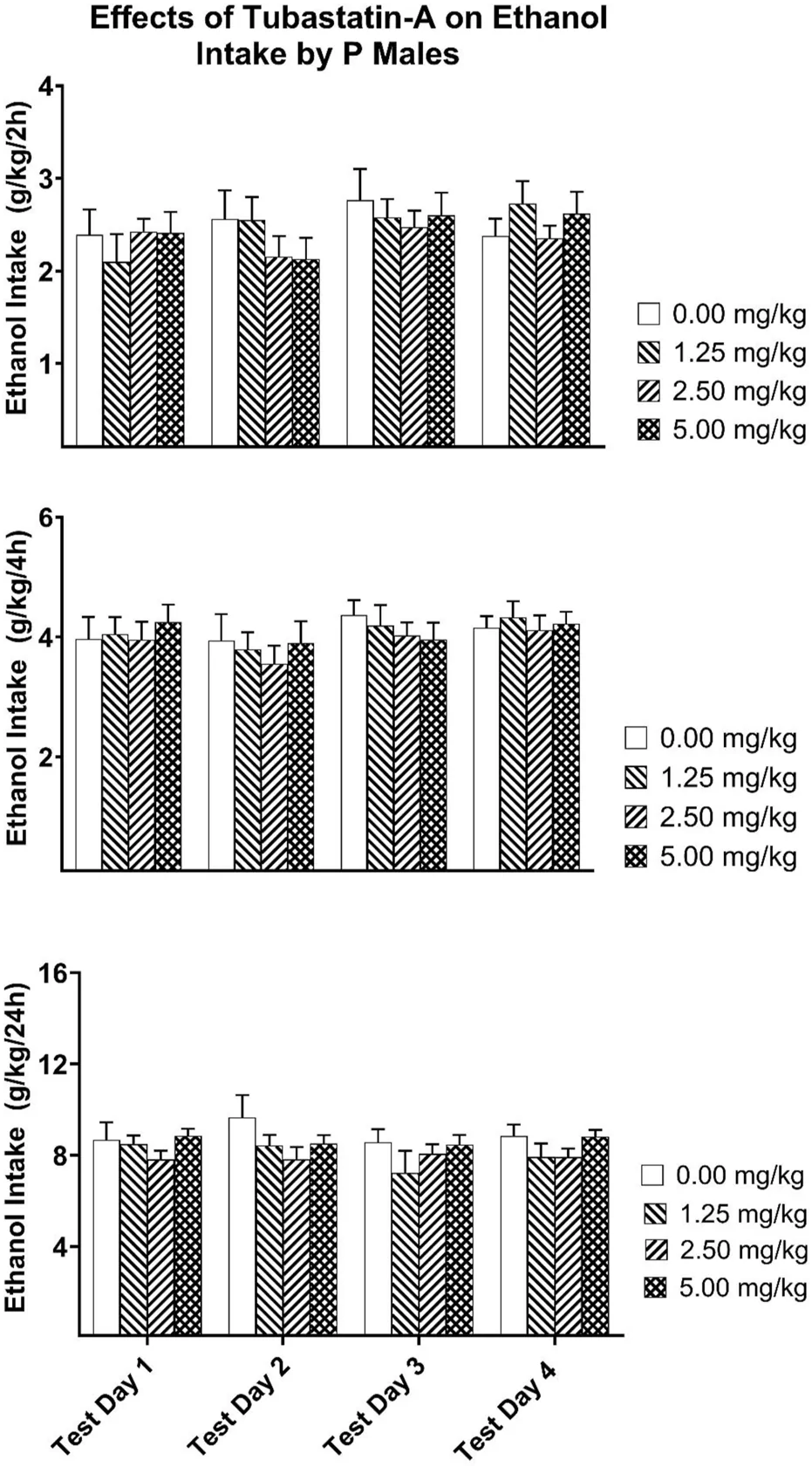
Depicts the mean (±SEM) ethanol intake (g/kg) in male P rats at (Upper Panel) 2-h, (Middle Panel) 4-h, and (Lower Panel) 24-h time points following i. p. administration 0, 1.25, 2.50, 5.00 mg/kg of tubastatin-A in male P rats.

#### HAD1 rats

For the HAD1 rats, the Dose × Test Day × Test Time mixed ANOVA revealed a significant Test Day × Test Time [F(6,210) = 2.310, p = 0.035; partial eta-squared = 0.062 with a power of 0.794] interaction (Figure 7). Analysis of the two-, four-, and 24-h ethanol intake data did not reveal any significant Dose effects.

**FIGURE 7:**
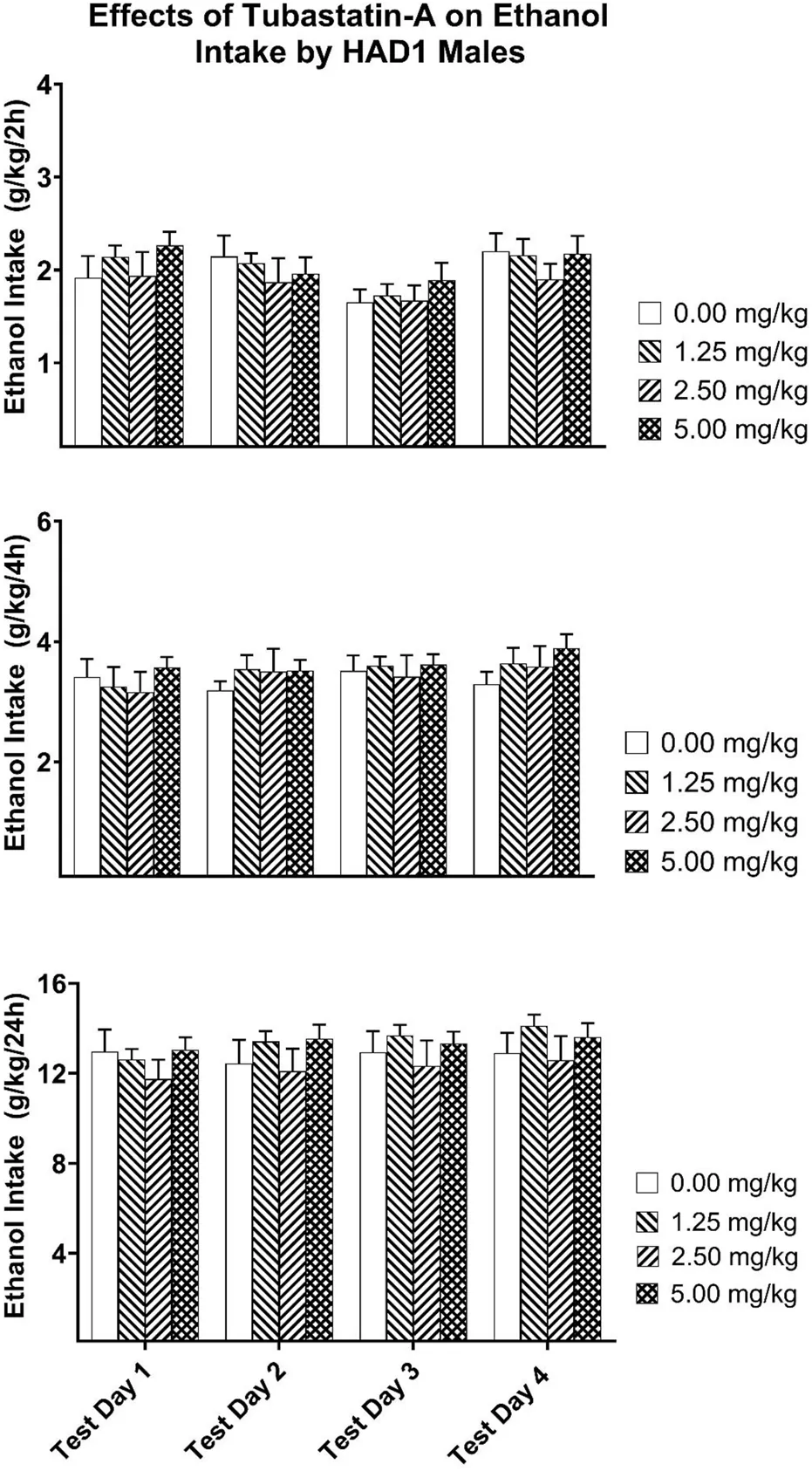
Depicts the mean (±SEM) ethanol intake (g/kg) in male HAD1 rats at (Upper Panel) 2-h, (Middle Panel) 4-h, and (Lower Panel) 24-h time points following i. p. administration 0, 1.25, 2.50, 5.00 mg/kg of tubastatin-A in male HAD1 rats.

### Effects of tubastatin-A on water intake

#### P rats

For the P rats (Supplementary Figure S5), the Dose x Test Day x Test Time mixed ANOVA revealed no significant Dose effects. Analysis of the two- and twenty-four water intake data revealed no significant Dose effects (Supplementary Figure S5). Analysis of the 4-h water intake revealed a significant Dose [F(3,35) = 5.139, p = 0.005; partial eta-squared 0.306, with a power of 0.891] main effect.

#### HAD1 rats

For the HAD1 rats (Supplementary Figure S6), the Dose × Test Day x Test Time mixed ANOVA revealed a significant Dose × Test Time [F(6,72) = 7.531, p < 0.001; partial eta-squared = 0.386, with a power of 0.999] interaction as well as a Dose [F(3,36) = 3.592, p = 0.023; partial eta-squared = 0.230, with a power of 0.745] main effect (Supplementary Figure S6). Analysis of the two- and 4-h water intake data revealed no significant Dose effects. Analysis of the 24-h water intake revealed a significant Dose [F(3,36) = 5.945, p = 0.002; partial eta-squared = 0.331, with a power of 0.934] main effect.

### Effects of tubastain-A on food intake and body weight

For the P (Supplementary Figure S11) and HAD1 (Supplementary Figure S12) rats, analysis of food intake and body weight data revealed no significant Dose effects.

## Discussion

The major findings of the present study were that the HDAC inhibitors (HDACi) entinostat, with selectivity for HDAC1 and HDAC3 (Figure 3; Table 1), and quisinostat, a second generation pan-HDACi (Figure 5; Table 1), reduced ethanol intake in HAD1, while only entinostat reduced ethanol intake in P rats (Figure 2; Table 1). The HDACi tubstatin-A, with selectivity for HDAC6, did not alter ethanol intake in HAD1 or P rats (Figures 6, 7; Table 1). In the current study, all doses of entinostat reduced ethanol intake in HAD1 rats both acutely (2-h and 4-h post-injection) and chronically (24-h). In contrast, the highest dose of entinostat was the most effective in reducing 24-h ethanol intake in P rats. These findings suggest that entinostat has a more robust effect in reducing ethanol drinking by HAD1 rats than by P rats. One possible explanation for the differences is the different genetic backgrounds of the rats, although both rat lines were selectively bred using the same criteria (>5 g of ethanol/kg bodyweight/day; Bell et al., 2016; Bell et al., 2017; McBride et al., 2014). P rats were selected from a closed colony of Wistar rats at Walter Reed Army Hospital and transferred to the Indiana University School of Medicine in Indianapolis, Indiana, United States (*cf.*, McBride and Li, 1998).

**TABLE 1:** The full results are summarized.

		P Rats						HAD1 Rats					
		2hr EtOH	4hr EtOH	24hr EtOH	2hr Water	4hr Water	24hr Water	2hr EtOH	4hr EtOH	24hr EtOH	2hr Water	4hr Water	24hr Water
Entinostat	1.25 mg/kg	–	–	–	–	–	↑ Day 2, 4	↓ Day 1, 2, 3, 4, 5	↓ Day 1, 2, 3, 4, 5	↓ Day 1, 2, 3, 4, 5	↑ Day 4	↑ Day 3, 4	↑ Day 1, 2, 3, 4
	2.50 mg/kg	↓ Day 3	↓ Day 2, 3, 5	↓ Day 2, 3, 5	↑ Day 3	–	↑ Day 3, 4	↓ Day 2, 3, 4, 5	↓ Day 2, 3, 4, 5	↓ Day 1, 2, 3, 4, 5	–	–	↑ Day 2, 3, 4
	5.00 mg/kg	–	↓ Day 3	↓ Day 1, 2, 3, 5	–	–	↑ Day 4	↓ Day 1, 2, 3, 4, 5	↓ Day 1, 2, 3, 4, 5	↓ Day 1, 2, 3, 4, 5	↑ Day 2, 3	↑ Day 1, 2	↑ Day 1, 2, 3, 4
Quisinostat	0.50 mg/kg	–	–	–	–	–	–	↓ Day 3, 4	↓ Day 4	↓ Day 4	–	–	–
	1.00 mg/kg	–	–	–	–	–	–	↓ Day 3, 4	↓ Day 4	↓ Day 4	–	–	–
	2.00 mg/kg	–	–	–	–	–	↑ Day 1, 4	↓ Day 1, 3, 4	↓ Day 3, 4	↓ Day 1, 2, 3, 4	↑ Day 2, 4	↑ Day 1, 2, 3, 4	↑ Day 1, 3, 4
Tubastatin-A	1.25 mg/kg	–	–	–	–	–	–	–	–	–	–	–	–
	2.50 mg/kg	–	–	–	–	–	–	–	–	–	–	–	–
	5.00 mg/kg	–	–	–	–	–	–	–	–	–	–	–	–

Entinostat, quisinostat, and tubastatin-A’s effects on ethanol and water intake in P and HAD1 rats at 2-h, 4-h, and 24-h time points. The downward arrows (↓) indicate ethanol intake was decreased. The upward arrows (↑) indicate that water intake was increased. The minus sign (─) indicates no effect on ethanol or water intake.

In contrast, HAD1 rats were derived from N/NIH heterogeneous stock rats (derived from eight inbred strains [ACI, BN, BUF, F344, M520, MR, WKY, and WN]) at Indiana University School of Medicine in Indianapolis, Indiana, United States (*cf.*, Bell et al., 2012). Another possibility is entinostat’s higher binding specificity for HDAC1 (IC _50_ values = 190 nM) and HDAC3 (IC _50_ values = 420 nM), over other HDACs (Marks et al., 2011). This may have limited its effect on P rat drinking. It has been reported that P rats do not have innate differences in HDAC 1, 3, 4, 5, or six protein levels within the amygdala compared to their control non-alcohol preferring (NP) rats, but P rats have an innately higher level of HDAC2 compared to NP rats (Moonat et al., 2013). NP rats are the low alcohol-consuming counterpart from the bidirectional selection that yielded P rats (Bell et al., 2012; McBride and Li, 1998). Therefore, entinostat effects on P rats may be via inhibition of HDAC2 (binding specificity IC_50_ value = 1900 nM; Marks et al., 2011). No published work to date has examined HDAC levels in HAD1 rats, compared with their low alcohol consuming low-alcohol-drinking (LAD1) counterparts (c.f., Bell et al., 2012), or HDAC levels in other brain areas of P rats.

Our findings are in line with previous studies, which demonstrated that entinostat can reduce ethanol intake in rodents. For example, systemic administration of entinostat (also known as MS-275) can robustly reduce excessive ethanol intake in ethanol-dependent rats using an oral operant ethanol self-administration procedure while not having an effect in non-dependent rats (Simon-O'Brien et al., 2015). Jeanblanc et al. (2015) reported that intracerebroventricular administration of entinostat reduced operant ethanol self-administration, decreased motivation to consume ethanol, and blocked relapse. Even after it was discontinued, Entinostat effectively reduced reacquisition to ethanol self-administration in rats (Jeanblanc et al., 2015). This may be due to entinostat’s long half-life (i.e., 9.8 h in rats and >50 h in humans) (Yang et al., 2014; Kurmasheva et al., 2019).

Entinostat has also been shown to reduce binge-like ethanol drinking in mice without altering saccharin consumption at a dose as high as 20 mg/kg (Warnault et al., 2013). This indicates that entinostat can selectively decrease ethanol drinking without altering the intake of natural rewards. However, the current study demonstrated that the highest dose (5 mg/kg) reduced food intake in both HAD1 and P rats during the last 2 or 3 test days without altering body weight. Thus, repeated administration of entinostat may result in possible cumulative effects on food intake. In addition, there may be species-dependent effects that differ between mice and rats. Entinostat tended to increase water intake, which may be a compensatory effect on liquid intake.

Ethanol has a strong modulating effect on the neuroimmune system (Crews et al., 2015; Cui et al., 2014; Erickson et al., 2019). The overexpression of neuroinflammatory factors observed following excessive ethanol consumption has been postulated to be a critical biological link between excessive ethanol consumption and the development of AUD (Crews et al., 2015; Cui et al., 2014; Erickson et al., 2019). Moreover, previous work from our laboratories has demonstrated that treatments with anti-neuroinflammatory agents can attenuate ethanol intake in HAD1 and P rats (ibudilast: Bell et al., 2015; PDE4 modulators; Franklin et al., 2015). Inhibition of HDACs can also reduce neuroinflammatory responses (Kannan et al., 2013; Patnala et al., 2017; Suliman et al., 2012; Xia et al., 2017). HDAC1 and HDAC3, like other Class I HDACs, are involved in innate immunity and cytokine production (Daskalaki et al., 2018; Kouzarides, 2007; Shakespear et al., 2011). Durham et al. (2017) reported that treatment with entinostat or siRNA knockdown of HDAC1 can reduce cytokine expression [i.e., tumor necrosis factor-α (TNF)-α and interleukin 6 (IL-6)] in microglia activated with lipopolysaccharide (LPS). Inhibition of HDAC3 has also been reported to reduce LPS-stimulated toll-like receptors in microglia, attenuate TNF-α and IL-6 production, and partially inhibit signal transducer and activator of transcription 3 (STAT3) and STAT5 as well (Xia et al., 2017). The latter are activated by cytokines and growth factors (e.g., Xia et al., 2017). Taken together, this suggests that entinostat’s ability to reduce ethanol intake in HAD1 and P rats may partly be due to its ability to inhibit inflammatory responses.

Quisinostat is a second-generation Class I and Class II HDAC inhibitor (Gatla et al., 2019), a good brain penetrant (Lo Cascio et al., 2023) with a half-life of about an hour in mice (Lo Cascio et al., 2023), and 8.8-h half-life in humans (Venugopal et al., 2013). At subnanomolar concentrations, it is specific for HDAC1 (IC _50_ values = 0.1 nM) and HDAC2 (IC _50_ values = 0.3 nM) (Lo Cascio et al., 2023), with ≥14-fold selectivity against the other Class I HDACs (i.e., HDAC3 [IC _50_ values = 4.8 nM] and HDAC 8 [IC _50_ values = 4.26 nM] as well the Class II HDACs; Arts et al., 2009). Compared with entinostat, quisinostat was not as effective in reducing HAD1 rats’ ethanol intake at the lowest and middle doses. Only the highest dose of quisinostat effectively decreased HAD1 rats' 24-h ethanol intake on all test days, with the greatest decrease on the third and fourth days. For the 2-h and 4-h drinking time points, quisinostat only effectively reduced ethanol intake on the third and fourth test days.

Interestingly, quisinostat did not significantly alter the P rats’ ethanol intake at any time point. Like entinostat, the highest dose of quisinostat tended to increase water intake suggesting a possible compensatory effect on liquid intake. However, HAD1 and P rats’ food intake was significantly reduced during the last 2 to 3 test days. To our knowledge, this is the first study to examine quisinostat’s effects on ethanol intake.

As previously mentioned, chronic ethanol exposure is associated with an overexpression of class I HDACs, particularly HDAC2, in limbic brain regions (i.e., hippocampus and amygdala). Therefore, it is possible that quisinostat’s attenuating effects on ethanol intake may, in part, be via inhibition of HDAC2 activity. However, Quisinostat was not effective in reducing P rats' ethanol drinking even though it has been reported that P rats have innately higher nuclear HDAC2 activity in the central and medial amygdala compared with their NP counterparts (Moonat et al., 2013). In addition, these authors reported that small interfering RNA (siRNA) knock-down of HDAC2 expression in the central nucleus of the amygdala (CeA) of P rats corrected ethanol-induced deficits by attenuating anxiety-like behaviors and inhibited ethanol-drinking behaviors in P rats (Moonat et al., 2013). A subsequent study found that trichostatin-A (TSA) can decrease HDAC2 levels as well as correct deficits in brain-derived neurotrophic factor, activity-regulated cytoskeleton-associated protein, and neuropeptide Y expression levels within the CeA of P rats (Sakharkar et al., 2014). It is noteworthy that this same trio of genes/proteins has been implicated in the development of AUD (Pandey et al., 2017).

Similar to pre-clinical research, HDAC2 expression in the amygdala was reported to be higher in the post-mortem brains of AUD subjects compared with control subjects (De Carvalho et al., 2021). An *in vitro* study revealed that ethanol exposure of a human neuronal cell line (SK-N-MC) upregulates gene and protein expression levels of HDAC2 through mechanisms involving oxidative stress, which often leads to inflammation (Agudelo et al., 2011). Moreover, TSA inhibition of HDAC2 gene and protein expression decreased reactive oxygen species production (Agudelo et al., 2011). Furthermore, TSA inhibited the effects following ethanol exposure of all class I HDACs (Agudelo et al., 2016). Collectively, these findings indicate that ethanol modulates class I HDAC activity and inhibition of class I HDACs may be useful for the treatment of AUD.

Quisinostat’s ineffectiveness in reducing P rat’s ethanol intake may be due to the sample size of five. However, the significant findings for water and food intake in P rats suggest against this conjecture. Another possibility is that a higher dose of quisinostat may be needed to reduce ethanol intake in P rats.

Tubastatin-A is highly specific for HDAC6 as a Class IIb HDAC with an IC50 of 15 nM for HDAC 6 (Butler et al., 2010). This was the only epigenetic compound tested in the current study that did not alter ethanol intake in HAD1 or P rats. This suggests HDAC6 may not regulate ethanol intake, at least in HAD1 and P rats. HDAC6 substrates, such as tubulin, heat shock proteins, and cortactin (Shen et al., 2020), are known to be affected by alcohol consumption (Azizov et al., 2023; Labisso et al., 2018; Mandrekar et al., 2008). The potential ineffectiveness of tubastatin-A could be due to alcohol altering different classes of the HDAC6 substrates than those targeted by tubastatin-A. Despite its low brain penetrance (Kozikowski et al., 2019) and a plasma half-life between 1 and 2 h in mice (Jochems et al., 2014; Shen et al., 2020), higher doses of tubastatin-A have shown promise in other animal disease models (e.g., Alzheimer’s disease, Parkinson’s disease, cardiac and pulmonary diseases; Shen et al., 2020). Therefore, another possible explanation is that a higher dose of tubastatin-A may be needed to reduce ethanol intake in rats.

Interestingly, López-Moreno et al. (2015) using RT qPCR reported that ethanol operant self-administration did not alter *HDAC6* gene expression in rats, but acute ethanol intoxication in humans increased *HDAC6* gene expression in peripheral blood samples. However, *Hdac6* mRNA levels were found to be increased in the central and basolateral amygdala in ethanol-dependent male rats compared to non-dependent male rats (De Carvalho et al., 2021; Repunte-Canonigo et al., 2015). Moreover, a recent clinical study reported an increase in *HDAC6* levels in the amygdala of AUD individual’s post-mortem brain tissue (De Carvalho et al., 2021). Inhibition of HADC6 has also been shown to attenuate LPS-induced inflammation markers in macrophages such as TNF-α, IL-1β, and IL-6 (Zhang et al., 2019). Therefore, further research is warranted to examine HDAC6 involvement in AUD as well as examine the effects of other HDAC6 inhibitors.

The current study does have some limitations. One limitation is that we did not examine female P and HAD1 rats. It is possible that female rats may respond differently to the HDAC inhibitors compared to male rats. The current study also did not examine whether the attenuating effects of the HDAC inhibitors were specific to ethanol drinking or whether they would reduce other reward drinking solutions, such as sucrose. Another limitation is that we did not investigate the effects of alcohol on HDAC genes and proteins across the different alcohol models, nor did we explore how the various HDAC inhibitor treatments might modify the effects of alcohol on HDAC gene and protein levels. Therefore, future studies should focus on assessing potential sex differences with different HDAC inhibitors and other rewards, as well as measuring gene and protein levels before and after HDAC inhibitor administration.

## Conclusion

In conclusion, the current study indicated that entinostat effectively reduced ethanol intake in two genetic rat models selected for high ethanol consumption, while quisinostat had a robust effect, it was effective in only one of these genetic rat models. Thus, genetic background may influence the effectiveness of certain HDAC inhibitors in reducing ethanol consumption. Finally, this study provides further evidence that targeting Class I HDACs may be an effective treatment for AUD.

## Data Availability

The raw data supporting the conclusions of this article will be made available by the authors, without undue reservation.
