# Supplementary material for: Effects of entinostat, quisinostat, and tubastatin-A on alcohol consumption in male high ethanol consuming rats
Source: Front Epigenet Epigenom. 2025 Feb 3;2:1503093. doi: 10.3389/freae.2024.1503093 (PMC13632490; doi:10.3389/freae.2024.1503093)
Supplement: Supplementary file 1 [file Supplementaryfile1.docx]

**SUPPLEMENTAL FIGURE LEGEND**

Figure S1. Depicts the mean (± SEM) water intake (ml) at (Upper Panel) 2-hour, (Middle Panel) 4-hour, and (Lower Panel) 24-hour time points following i.p. administration 0, 1.25, 2.5, 5 mg/kg of entinostat. *(p < 0.05) Indicates that entinostat significantly increased water intake compared to control in male P rats.

Figure S2. Depicts the mean (± SEM) water intake (ml) at (Upper Panel) 2-hour, (Middle Panel) 4-hour, and (Lower Panel) 24-hour time points following i.p. administration 0, 1.25, 2.5, 5 mg/kg of entinostat. *(p < 0.05) Indicates that entinostat significantly increased water intake compared to control in male HAD1 rats.

Figure S3. Depicts the mean (± SEM) water intake (ml) at (Upper Panel) 2-hour, (Middle Panel) 4-hour, and (Lower Panel) 24-hour time points following i.p. administration 0, 0.5, 1.0, 2.0 mg/kg of quisinostat. *(p < 0.05) Indicates that quisinostat significantly increased water intake compared to control in male P rats.

Figure S4. Depicts the mean (± SEM) water intake (ml) at (Upper Panel) 2-hour, (Middle Panel) 4-hour, and (Lower Panel) 24-hour time points following i.p. administration 0, 0.5, 1.0, 2.0 mg/kg of quisinostat. *(p < 0.05) Indicates that quisinostat significantly increased water intake compared to control in male HAD1 rats.

Figure S5. Depicts the mean (± SEM) water intake (ml) at (Upper Panel) 2-hour, (Middle Panel) 4-hour, and (Lower Panel) 24-hour time points following i.p. administration 0, 1.25, 2.5, 5 mg/kg of tubastatin-A in male P rats.

Figure S6. Depicts the mean (± SEM) water intake (ml) at (Upper Panel) 2-hour, (Middle Panel) 4-hour, and (Lower Panel) 24-hour time points following i.p. administration 0, 1.25, 2.5, 5 mg/kg of tubastatin-A in male HAD1 rats.

Figure S7. Depicts the mean (± SEM) body weight (g) and food intake (g) at 24-hour time point following i.p. administration 0, 1.25, 2.5, 5 mg/kg of entinostat. (Upper Panel) P rats body weight (Lower Panel) P rats food intake *(p < 0.05) Indicates that entinostat significantly reduced body weight or food intake compared to control in male P rats.

Figure S8. Depicts the mean (± SEM) body weight (g) and food intake (g) at 24-hour time point following i.p. administration 0, 1.25, 2.5, 5 mg/kg of entinostat. (Upper Panel) HAD1 rat's Body weight and (Lower Panel) HAD1 rat's food intake. *(p < 0.05) Indicates that entinostat significantly reduced body weight or food intake compared to control in male HAD1 rats.

Figure S9. Depicts the mean (± SEM) body weight (g) and food intake (g) at 24-hour time point following i.p. administration 0, 0.5, 1.0, 2.0 mg/kg of Quinsinostat. (Upper Panel) P rat's Body weight and (Lower Panel) P rat's food intake. *(p < 0.05) Indicates that Quinsinostat significantly reduced Body weight and/or food intake compared to control in male P rats.

Figure S10. Depicts the mean (± SEM) body weight (g) and food intake (g) at 24-hour time point following i.p. administration 0, 0.5, 1.0, 2.0 mg/kg of quinsinostat. (Upper Panel) HAD1 rat's Body weight and (Lower Panel) HAD1 rat's food intake *(p < 0.05) Indicates that quinsinostat significantly reduced body weight and/or food intake compared to control in male HAD1 rats.

Figure S11. Depicts the mean (± SEM) body weight (g) and food intake (g) at 24-hour time point following i.p. administration 0, 1.25, 2.50, 5.00 mg/kg of tubastatin-A. (Upper Panel) P rats' body weight and (Lower Panel) P rats' food intake.

Figure S12. Depicts the mean (± SEM) body weight (g) and food intake (g) at 24-hour time point following i.p. administration 0, 1.25, 2.50, 5.00 mg/kg of tubastatin-A. (Upper Panel) HAD1 rats' body weight and (Lower Panel) HAD1 rats' food intake.
